# Comparisons of Long-Term Survival and Safety of Haploidentical Hematopoietic Stem Cell Transplantation After CAR-T Cell Therapy or Chemotherapy in Pediatric Patients With First Relapse of B-Cell Acute Lymphoblastic Leukemia Based on MRD-Guided Treatment

**DOI:** 10.3389/fimmu.2022.915590

**Published:** 2022-06-06

**Authors:** Guanhua Hu, Yifei Cheng, Yingxi Zuo, Yingjun Chang, Pan Suo, Yueping Jia, Aidong Lu, Yu Wang, Shunchang Jiao, Longji Zhang, Yuqian Sun, Chenhua Yan, Lanping Xu, Xiaohui Zhang, Kaiyan Liu, Yu Wang, Leping Zhang, Xiaojun Huang

**Affiliations:** ^1^ Peking University People’s Hospital, Peking University Institute of Hematology, National Clinical Research Center for Hematologic Disease, Beijing Key Laboratory of Hematopoietic Stem Cell Transplantation, Peking-Tsinghua Center for Life Science, Research Unit of Key Technique for Diagnosis and Treatment of Hematologic Malignancies, Chinese Academic of Medical Sciences, Beijing, China; ^2^ Department of Pediatrics, Peking University People’s Hospital, Peking University, Beijing, China; ^3^ Department of Immunotherapy, Beijing Yongtai Reike Biotechnology Company Ltd., Beijing, China; ^4^ Department of Hematology, Chinese People Liberation Army (PLA) General Hospital, Beijing, China; ^5^ Department of Immunotherapy, Shenzhen Geno-immune Medical Institute, Shenzhen, China

**Keywords:** CAR-T therapy, B-cell acute lymphoblastic leukemia, pediatric, haploidentical hematopoietic stem cell transplantation, measurable residual disease (MRD)

## Abstract

Measurable residual disease (MRD) positivity before haploidentical hematopoietic stem cell transplantation (haplo-HSCT) is an independent prognostic factor in determining outcomes in patients with B-cell acute lymphoblastic leukemia (ALL). In this study, we conducted a parallel comparison of the efficacy and safety in patients with suboptimal MRD response after reinduction who underwent haplo-HSCT after chimeric antigen receptor T-cell (CAR-T) therapy or chemotherapy. Forty B-cell ALL patients who relapsed after first-line chemotherapy and with an MRD ≥0.1% after reinduction were analyzed. The median pre-HSCT MRD in the CAR-T group (*n* = 26) was significantly lower than that in the chemotherapy group (*n* = 14) (0.009% vs. 0.3%, *p* = 0.006). The CAR-T group exhibited a trend toward improved 3-year leukemia-free survival and a significantly improved 3-year overall survival compared to the chemotherapy group [71.8% (95% confidence interval (CI): 53.9–89.6) vs. 44.4% (95% CI: 15.4–73.4), *p* = 0.19 and 84.6% (95% CI: 70.6–98.5) vs. 40.0% (95% CI: 12.7–67.2), *p* = 0.008; respectively]. Furthermore, no increased risk of graft-versus-host disease, treatment-related mortality, or infection was observed in the CAR-T group. Our study suggests that CAR-T therapy effectively eliminates pre-HSCT MRD, resulting in better survival in the context of haplo-HSCT.

## Introduction

Relapse remains the main cause of mortality in childhood B-cell acute lymphoblastic leukemia (ALL); approximately 20% of patients fail to respond to chemotherapy after relapse (1), with a 5-year survival rate of 30% (2, 3). Allogeneic hematopoietic stem cell transplantation (allo-HSCT) is an effective treatment option for patients experiencing relapse after first-line chemotherapy (4). However, tumor load has been reported to be closely related to the outcomes after transplant; patients failed to achieve complete remission (CR) before HSCT, with a reported 3-year leukemia-free survival (LFS) rate of 21%–29%. Moreover, the importance of pre-HSCT measurable residual disease (MRD) status has gained appreciation in recent years and has shown significantly inferior outcomes in ALL patients with positive pre-MRD (5). Bader et al. have reported that the cumulative incidence of relapse for pre-MRD-negative, MRD < 0.01%, MRD <0.1% and ≥0.01%, and MRD ≥0.1% was 11%, 20%, 64%, and 54%, respectively (*p* < 0.001) (6). Thus, more effective therapies are required for patients experiencing relapse after first-line therapy for ALL.

Previous clinical trials have shown that refractory/relapse B-cell ALL patients receiving chimeric antigen receptor T-cell (CAR-T) therapy attained higher MRD-negative CR rates than those receiving conventional chemotherapy (6). However, studies on the efficiency and safety of CAR-T therapy compared with chemotherapy on pre-HSCT MRD eradication and survival based on MRD-guided treatment in the context of haploidentical HSCT (haplo-HSCT) are lacking. Therefore, in this study, we conducted a parallel comparison of the prognosis and treatment-related complications among relapse patients with suboptimal MRD response after reinduction chemotherapy who underwent haplo-HSCT from either CAR-T therapy or chemotherapy.

## Methods

### Patients

We included first relapse of B-cell ALL patients who had suboptimal MRD response after reinduction chemotherapy and underwent haplo-HSCT bridged from either CAR-T therapy or chemotherapy at Peking University People’s Hospital between April 2015 and April 2020. The exclusion criteria were as follows: (1) isolated extramedullary relapse; (2) previous transplantation and/or previous CAR-T therapy; (3) significant cardiovascular, hepatic, and renal dysfunction and active infections; (4) MRD < 0.1% after reinduction chemotherapy; and (5) patients followed by matched sibling donor transplantation (MSDT). Because the uniformity of treatment protocols can help to reduce the impact of different treatment protocols and most of the donors of allo-HSCT in our institute were haplo, 3 patients followed by MSDT were excluded. The study was approved by the Ethics Committee of Peking University People’s Hospital.

### Detection of MRD

A panel of eight antibody combinations, which included cCD3, mCD3, CD2, CD5, CD7, CD10, CD19, CD20, CD34, CD38, CD45, CD58, CD99, CD123, and cTDT, were used for MRD detection. The standardized assays and quality controls were consistent with those of previous reports (7, 8). Any MRD level was considered positive. MRD was assessed every month until the patient undergoes HSCT in this study.

### Treatment Protocol and Evaluation

All patients received a reinduction chemotherapy regimen (vincristine 1.5 mg/m2/day, days 1, 8, 15 and 22; idarubicin 8–10 mg/m2/day, days 1 and 8; cyclophosphamide 1,000 mg/m2/day, day 1; prednisone 60 mg/m2/day, days 1–28; and L-asparaginase 10,000 ug/m2/day, days 15–33) and risk-based intrathecal chemotherapy. After reinduction, morphologic response and multiparameter flow cytometry-MRD (FCM-MRD) were evaluated, and any level of MRD was considered positive.

In the chemotherapy group, consolidation chemotherapy regimens composed of HDMTX (methotrexate 2.5–3.5 g/m2/day, day 1; vincristine 1.5 mg/m2/day, day 1; with or without peg-aspargase 3750 ug/m2/day, day 3), HDAra-c (cytarabine 2 g/m2/day, days 1–3; idarubicin 8–10 mg/m2/day, days 2 and 3), and/or IFO (ifosfamide 1 g/m2/day, days 1–5; etoposide 100 mg/m2/day, days 3–5; vincristine 1.5 mg/m2/day, day 1) were given, and patients were subjected to HSCT as soon as they achieved MRD negativity. For patients who failed to achieve MRD negativity, the chemotherapy regimens and the time of processing to HSCT were chosen by the doctors based on the patient’s condition and treatment history.

CAR-T cells in this study were investigational products and conducted in the setting of clinical trials (www.clinicaltrials.gov as #NCT 03050190; www.chictr.org.cn as #ChiCTR-OPN-17013507). Anti-CD19 CAR-T cells constructed with 4-1BB or CD28 costimulatory domains were generated *via* a lentiviral vector from fresh leukapheresis material. Lymphodepleting chemotherapy, including fludarabine- and cyclophosphamide-based conditioning treatments, were administered prior to CAR-T infusion.

The conditioning regimen for haplo-HSCT was in accordance with previous reports (9, 10). For patients with extramedullary relapse, a total body irradiation (TBI)-based conditioning regimen was administered, which included TBI (770 Gy) on day −6, cyclophosphamide (1.8 g/m^2^/day) from days −5 to −4, simustine (250 mg/kg/day) on day −3, and antithymocyte globulin (ATG) (2.5 mg/kg/day) from days −5 to −2. For patients with isolated bone marrow relapse, the conditioning regimen included cytarabine (4 g/m^2^/day) from days −10 to −9, busulfan (3.2 mg/kg/day) from days −8 to −6, cyclophosphamide (1.8 g/m^2^/day) from days −5 to −4, simustine (250 mg/kg/day) on day −3, and ATG (2.5 mg/kg/day) from days −5 to −2. After the conditioning regimen, all patients received granulocyte colony-stimulating factor-mobilized, fresh, and unmanipulated (without T-cell depletion of graft *in vitro*) bone marrow cells plus peripheral blood stem cells (PBSCs) or PBSCs alone. The regimen for preventing graft-versus-host disease (GVHD) included cyclosporin A, mycophenolate mofetil, and short-term methotrexate.

### Study Endpoints and Definitions

The primary endpoints were the MRD response, LFS, and OS. The secondary endpoints were non-relapse mortality (NRM), acute GVHD (aGVHD), chronic GVHD (cGVHD), and complications after haplo-HSCT. Early marrow relapse was defined as relapse in <36 months from diagnosis, whereas late marrow relapse was defined as relapse in ≥36 months from diagnosis. CR2 was defined as bone marrow blasts <5%, neutrophils >1.0 × 10^9^/L, platelet count >100 × 10^9^/L, and extramedullary disease absence. LFS, overall survival (OS), non-relapse related mortality (NRM), engraftment, aGVHD, and chronic GVHD (cGVHD) were defined as described previously (9).

### Statistical Analysis

Enumeration data were compared using the chi-square test or Fisher’s exact test, whereas the Mann–Whitney *U* test was used for continuous variables. The Kaplan–Meier method was used to analyze LFS and OS. The competing risk model was used to analyze cumulative incidence, and Gray’s test was used to assess the differences between cumulative incidences in univariable analyses. Multivariate analysis was performed using the Cox proportional hazards regression model. All *p*-values were two-sided, and values <0.05 were considered statistically significant. The Statistical Program for Social Sciences (SPSS) software (version 23.0; SPSS Inc. Chicago, IL, USA) and R version 3.5.3 (R Foundation for Statistical Computing, Vienna, Austria) were used for data analysis.

## Results

### Patients

**Figure 1:**
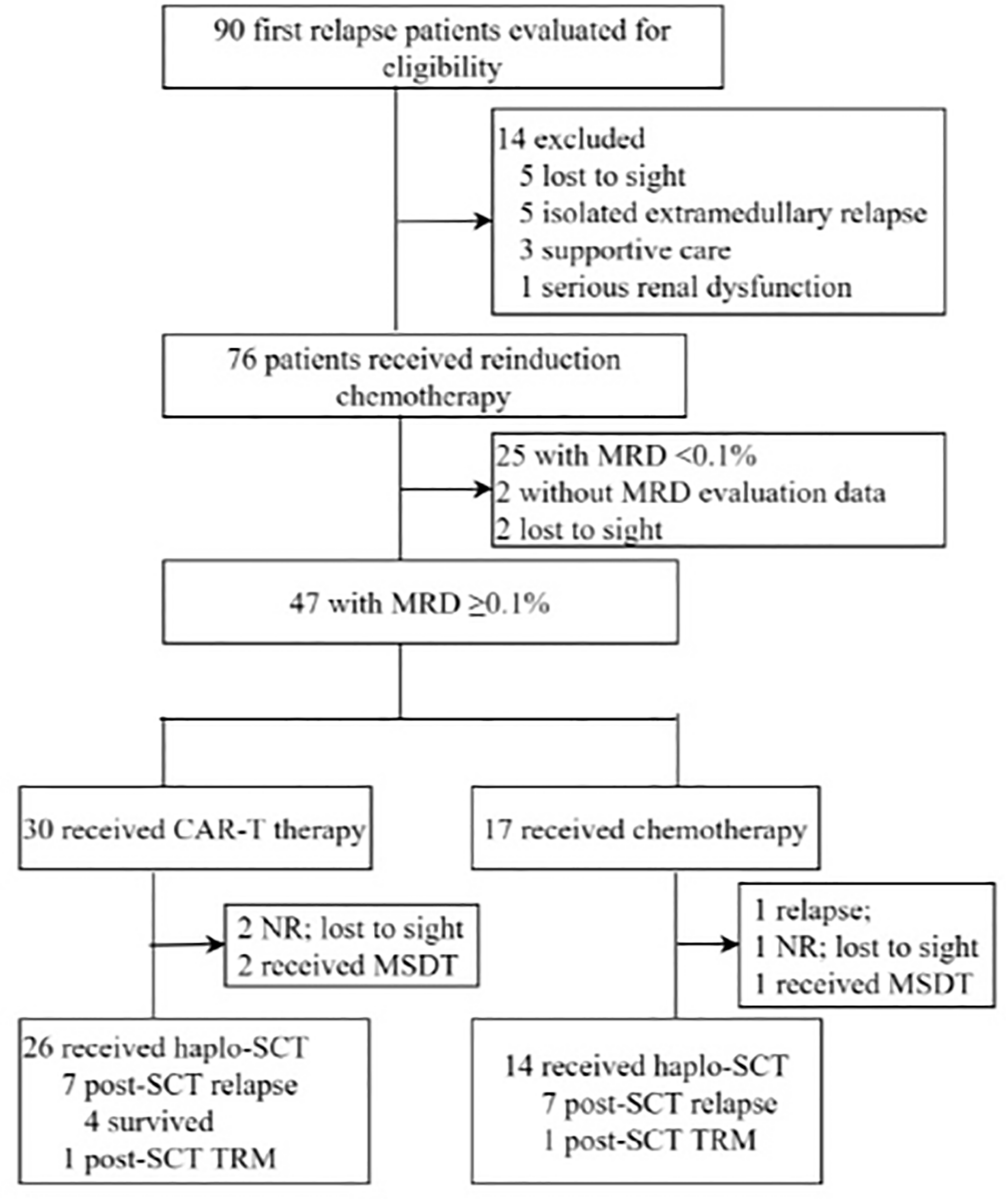
Diagram of patients enrolled in this study. CAR-T, chimeric antigen receptor T cells; CR, complete remission; HSCT, hematopoietic stem-cell transplant; MRD, measurable residual disease; MSDT, matched siblings donor transplantation; NR, non-remission; TRM, treatment-related mortality.

In this study, 90 first relapse B-cell ALL patients were treated, and 40 patients with MRD ≥ 0.1% after reinduction were analyzed (**Figure 1**). The median age at relapse was 9.2 years (2–18 years) (**Table 1**), and the median duration of CR1 to relapse diagnosis was 27.2 months (3–57 months), including 23 and 17 patients with early and late relapse, respectively. The main site of relapse was isolated bone marrow (*n* = 32, 80%). Four (10%) patients were Philadelphia chromosome-positive, and fourteen (35%) patients harbored high-risk molecular and cytogenetic features. The median levels of bone marrow blasts and MRD at relapse diagnosis were 49.2% (8%–96%) and 24.2% (4%–94%), respectively. Twenty-five (62.5%) patients achieved CR2 after reinduction.

**Table 1:** Characteristics of patients stratified by CAR-T and chemotherapy groups.

Characteristic	CAR-T group (*n* = 26)	Chemotherapy group (*n* = 14)	*p*
Age at relapse			
Median, years	9.5	9.0	0.706
Sex			
Female	12	5	0.747
Male	16	9	
Cytogenetic group Favorable	6	3	0.906
ETV6-RUNX1	3	3	
Hyperdiploid	3	–	
Unfavorable	8	7	0.310
Hypodiploid	–	1	
Complex karyotype	–	2	
E2A-PBX1	3	–	
BCR-ABL1	3	1	
Ph-like	–	1	
IKZF1 mutation	2	2	
Site of relapse			
Isolated bone marrow	22	11	0.679
Combined bone marrow and extramedullary	4	3	
Timing of relapse			
<36 months after diagnosis	16	7	0.521
≥36 months after diagnosis	10	7	
Bone marrow blasts after relapse median, %	40	56	0.125
MRD after relapse median, %	22	25	0.890
Disease status before HSCT			
NR	0	1	0.350
MRD-positive CR	5	4	0.505
MRD-negative CR	21	10	
MRD before HSCT median (range), %	0.009 (0–1.7)	0.304 (0–3.8)	0.006

CAR-T, chimeric antigen receptor T cells; CR, complete remission; HSCT, hematopoietic stem cell transplant; MRD, measurable residual disease; NR, non-remission.

In the CAR-T group, nine (34.6%) patients remained NR before CAR-T cell infusion. The median MRD level was 7.2% (0.07%–72%) before CAR-T cell infusion. The median dose of CAR-T cells was 4.04 × 10^6^/kg (0.35–6.51 × 10^6^/kg). After 1 month of infusion, all patients were in CR2. MRD-negative CR was achieved by twenty-three (88.5%) patients, whereas MRD-positive CR was achieved by three (11.5%) patients; the median MRD level was 0.005% (0%–0.17%). Cytokine release syndrome (CRS) of any grade was observed in fifteen (57.7%) patients, whereas severe CRS (grades 3 and 4) was observed in three (11.5%) patients. Neurological adverse events occurred in three (11.5%) patients, but no CAR-T-related mortality was observed.

In the chemotherapy group, six patients remained NR after reinduction, and the median MRD level was 7.6% (0.11%–44%) after reinduction. Before haplo-HSCT, patients in the chemotherapy group received 3.4 (1–6) cycles of chemotherapy. No chemotherapy-related mortality was observed.

### Response

Among the patients receiving haplo-HSCT, 39 (97.5%) achieved CR2 before HSCT, whereas 12 (30%) had positive pre-HSCT MRD; the median level of pre-HSCT MRD was 0.11% (0%–3.8%).

The median time from CAR-T therapy to haplo-HSCT was 56 days (30–90 days). CR2 status was attained before haplo-HSCT by 26 (100%) and 13 (92.8%) patients in the CAR-T and chemotherapy groups, respectively. Pre-HSCT MRD-negative CR was achieved by 21 (80.7%) and 10 (71.4%) patients in the CAR-T and chemotherapy groups, respectively. The median pre-HSCT MRD in the CAR-T group was 0.009%, which was significantly lower than that in the chemotherapy group (0.3%, *p* = 0.006).

### Long-Term Survival

Post-HSCT relapse occurred in 7 (26.9%) patients at a mean period of 12.4 months in the CAR-T group; among them, three patients died of relapse, and four patients responded to salvage therapy (two survived by HSCT, one by CAR-T, and one by chemotherapy plus donor leukocyte infusion). In the chemotherapy group, post-HSCT relapse occurred in seven (50%) patients at a mean period of 10.5 months, and seven patients died of relapse. In addition, two patients (one from each group) died of NRM.

**Figure 2:**
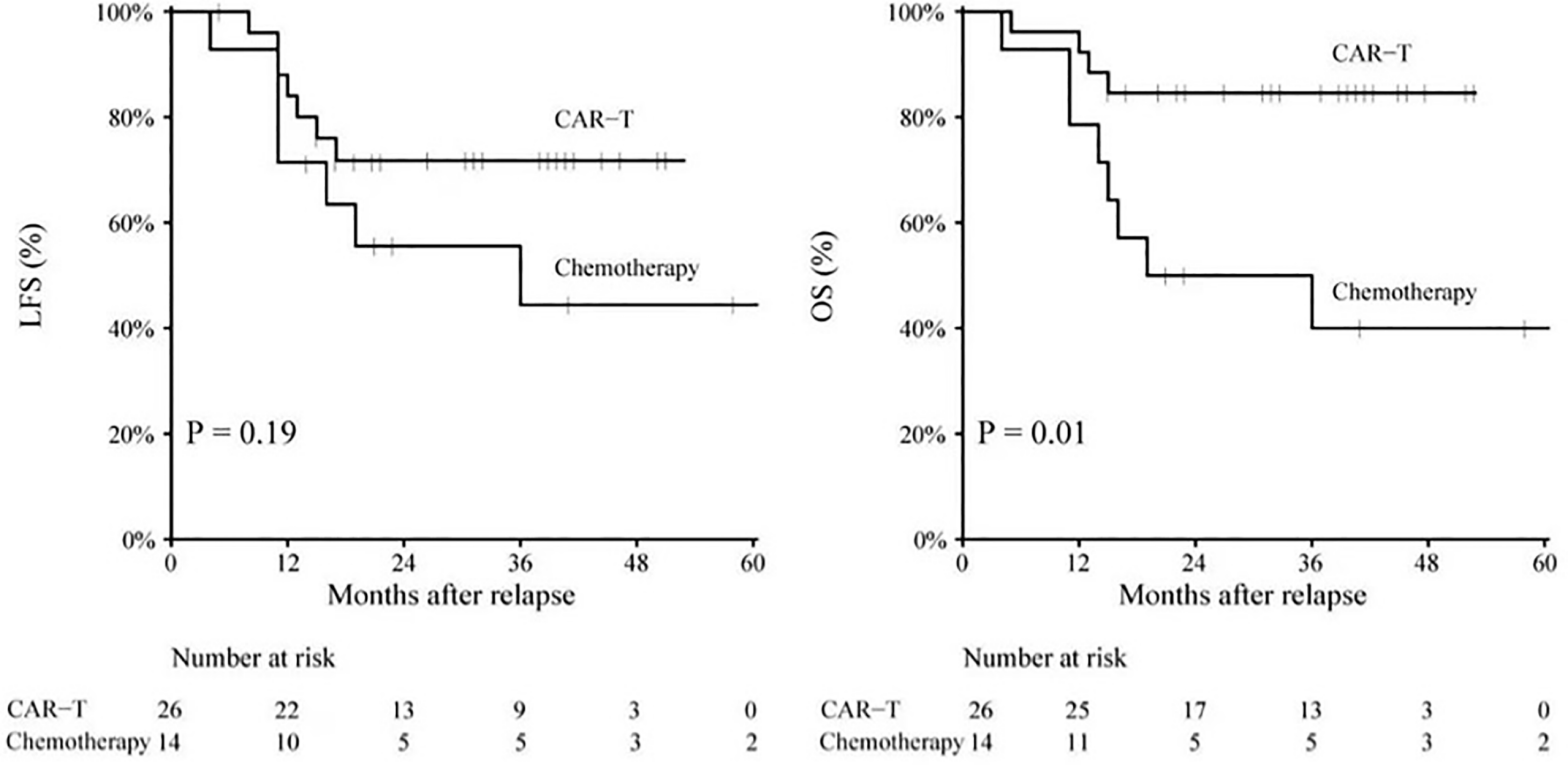
LFS rate and OS rate for patients in the CAR-T group and patients in the chemotherapy group. CAR-T, chimeric antigen receptor T cells; LFS, leukemia-free surival; OS, overall survival.

The mean follow-up time was 37.3 years for survived patients. Overall, 3-year probabilities of LFS and OS were 61.2% [95% confidence index (CI): 44.5–77.8] and 68.2% (95% CI: 52.9–83.5). The CAR-T group had a trend toward improved 3-year LFS compared to the chemotherapy group [71.8% (95% CI: 53.9–89.6) vs. 44.4% (95% CI: 15.4–73.4), *p* = 0.19] (**Figure 2**). The CAR-T group had a significantly improved 3-year OS compared to the chemotherapy group [84.6% (95% CI: 70.6–98.5) vs. 40.0% (95% CI: 12.7–67.2), *p* = 0.008].

Among all patients, aged <10 years, isolated bone marrow relapse, pre-HSCT MRD positivity, and non-CAR-T therapy were risk factors for inferior survival (**Table 2**). In the CAR-T group, the factors of isolated bone marrow relapse and pre-HSCT MRD positivity may have been abrogated by CAR-T therapy; the 3-year LFS and OS of patients with isolated bone marrow relapse were comparable with those of combined relapse [64.6% (95% CI: 43.4–85.7) vs. 100%, *p* = 0.145] [82.0% (95% CI: 65.1–98.8) vs. 100%, *p* = 0.310], and the 3-year LFS and OS of patients with pre-HSCT MRD positivity were comparable with those of patients with pre-HSCT MRD negativity [75.0% (95% CI: 32.4–117.5) vs. 71.7% (95% CI: 52.1–91.3), *p* = 0.853] [60.0% (95% CI: 17.1–102.1) vs. 90.5% (95% CI: 77.9–103.0), *p* = 0.067]. However, in the CAR-T group, patients aged <10 years had an inferior 3-year OS than those aged ≥10 years [71.4% (95% CI: 47.6–95.1) vs. 94.5% (95% CI: 87.9–113.0), *p* = 0.049], but the 3-year LFS of patients aged <10 years was comparable with that of patients aged ≥10 years [61.5% (95% CI: 35.1–87.9) vs. 82.5% (95% CI: 60.3–104.6), *p* = 0.220].

**Table 2:** Clinical characters and their association with survival.

	Patients(*n*)	Log-rank analysis				Univariate Cox regression analysis			
		LFS (95% CI)	*p*	OS (95% CI)	*p*	HR (95% CI) for LFS	*p*	HR (95% CI) for OS	*p*
Age, years									
1–9	23	47.4 (26.8–31.1)	0.046	54.4 (33.4–75.4)	0.041	1	0.061	1	0.506
10–18	17	73.7 (51.1–96.2)		78.7 (56.7–100.1)		0.34 (0.11–1.05)		0.65 (0.17–2.34)	
Sex									
Male	27	53.1 (33.1–73.1)	0.537	59.3 (39.3–79.3)	0.501	1	0.546	1	0.078
Female	13	69.2 (44.1–94.3)		75.2 (50.5–99.8)		0.71 (0.23–2.19)		0.31 (0.08–1.14)	
Cytogenetic group									
Non-unfavorable	25	67.3 (48.6–85.9)	0.186	69.5 (50.5–88.5)	0.661	1	0.201	1	0.663
Unfavorable	15	42.7 (15.3–70.1)		57.1 (30.2–83.9)		1.89 (0.71–5.06)		1.27 (0.43–3.79)	
Site of relapse									
Isolated bone marrow relapse	33	46.8 (27.9–65.6)	0.018	54.0 (34.6–73.4)	0.035	1	0.159	1	0.203
Combined bone marrow and extramedually	7	100		100		0.03 (0–3.89)		0.03 (0–6.51)	
Timing of relapse									
<36 months	23	48.4 (25.7–71.1)	0.211	58.0 (36.0–79.9)	0.399	1	0.228	1	0.406
≥36 months	17	70.1 (48.1–92.0)		73.1 (50.4–95.8)		0.52 (0.18–1.50)		0.61 (0.18–1.97)	
Response of reinduction									
CR	25	64.8 (44.4–85.2)	0.113	70.7 (50.1–91.3)	0.118	1	0.128	1	0.131
NR	15	46.7 (21.4–71.9)		53.3 (28.0–78.6)		2.15 (0.80–5.75)		2.32 (0.78–6.93)	
Pre-HSCT treatment									
CAR-T therapy	26	71.8 (53.9–89.6)	0.190	84.6 (70.6–98.5)	0.008	1	0.176	1	0.039
Chemotherapy	14	44.4 (15.4–73.4)		40.0 (12.7–67.2)		1.97 (0.74–5.25)		3.25 (1.06–9.94)	
Pre-HSCT MRD									
Positive	12	33.3 (6.6–60.0)	0.041	37.0 (8.4–65.6)	0.012	1	0.053	1	0.020
Negative	28	71.2 (54.3–88.1)		77.7 (61.8–93.6)		2.64 (0.98–7.03)		3.68 (1.23–11.03)	

CAR-T, chimeric antigen receptor T cells; CI, confidence index, CR, complete remission; HSCT, hematopoietic stem cell transplant; LFS, leukemia-free survival; MRD, measurable residual disease; NR, non-remission; OS, overall survival.

### Engraftment After Haplo-HSCT

The median dose of infused MNC and CD34 in the CAR-T group was comparable with that in the chemotherapy group [9.3×10^8^/kg (6.2–12.9×10^8^/kg) vs. 9.4×10^8^/kg (6.6–12.2×10^8^/kg), *p* = 0.863] [2.8×10^6^/kg (1.3–7.4×10^6^/kg) vs. 2.5×10^6^/kg (1.1–4.5×10^6^/kg), *p* = 0.519]. All patients achieved neutrophil engraftment, and the mean duration of neutrophil engraftment in the CAR-T group was comparable with that in the chemotherapy group [13 days (11–21 days) vs. 13 days (11–17 days), *p* = 0.525]. The mean duration of platelet engraftment in the CAR-T group was 16 days (8–34 days), which was comparable with that in the chemotherapy group [15 days (10–43 days), *p* = 0.891].

### Complications After Haplo-HSCT

The cumulative 100-day incidences of aGVHD grades II–IV and III and IV in the CAR-T group were similar to those in the chemotherapy group [26% (95% CI: 21%–31%) vs. 23% (95% CI: 18%–28%), *p* = 0.303 and 7% (95% CI: 5%–9%) vs. 5% (95% CI: 4%–6%), *p* = 0.723; respectively] (**Table 3**). The cumulative 3-year incidences of total and severe cGVHD in the CAR-T group were also similar to those in the chemotherapy group [53% (95% CI: 36%–70%) vs. 50% (95% CI: 41%–59%), *p* = 0.623 and 11% (95% CI: 8%–14%) vs. 10% (95% CI: 5%–15%), *p* = 0.876; respectively].

**Table 3:** Complications of patients stratified by CAR-T and chemotherapy groups.

Complications	CAR-T group (*n* = 26)	Chemotherapy group (*n* = 14)
II–IV aGVHD	8	3
Total cGVHD	16	9
Cytomegalovirus infection Epstein–Barr virus infection	22 3	11 2
TA-TMA	1	0
TRM	1	1

CAR-T, chimeric antigen receptor T cells; GVHD, graft-versus-host disease; TA-TMA, transplantation-association thrombotic microangiopathy; TRM, treatment-related mortality.

The 100-day incidences of cytomegalovirus and Epstein–Barr virus reactivation in the CAR-T group were comparable with those in the chemotherapy group [73.1% (95% CI: 52.8%–93.4%) vs. 65.0% (95% CI: 48.9%–81.1%), *p* = 0.75 and 11.5% (95% CI: 8.7%–14.3%) vs. 14.2% (95% CI: 10.1%–18.3%), *p* = 0.64; respectively]. One patient in the CAR-T group was diagnosed as transplantation-association thrombotic microangiopathy, which was not found in the chemotherapy group. One patient in the CAR-T group died of gastrointestinal hemorrhage and intracranial infection, and one patient in the chemotherapy group died of severe pneumonia and respiratory failure.

## Discussion

Although the prognosis of pediatric ALL has been proven, >85% of patients survived without relapse (11). However, survival following relapse is dismal; the Children’s Oncology Group has reported that the 5-year OS rate for relapse patients is 36% (12), and the 10-year OS for children treated in the ALL-REZ BFM 90 trial was 36% (13). Multiple studies have demonstrated that the interval between remission and relapse and the site of relapse are important predictors of outcome (14–16). Moreover, MRD response to reinduction has been defined as the strongest prognostic factor for relapsed ALL; the ALL-REZ BFM P95/96 trial showed that MRD <0.1% after reinduction predicted a 10-year event-free survival (EFS) of >70% and MRD ≥0.1% after reinduction predicted a 10-year EFS of <20% with conventional chemotherapy (17). Therefore, an MRD-based strategy to intensify treatment with allo-HSCT in patients with MRD ≥0.1% after reinduction was implemented in the ALL-REZ BFM 2002 trial and an improved outcome (8-year EFS, 64% vs. 18% in the historical control) was observed (18). However, some studies have suggested that pre-HSCT MRD positivity is associated with subsequent relapse and poor survival (8, 19). The International BFM Study Group reported the first prospective study to evaluate the effect of pre-HSCT MRD status and found that the 4-year EFS was the highest at 64% for undetectable MRD and 48% for MRD <0.01%; a multivariate Cox regression model confirmed that pre-HSCT MRD positivity (>0.01%) was the only independent prognostic factor for relapsed ALL (6). We found the same results in this study; patients with pre-HSCT MRD positivity had significantly lower 3-year LFS than those with pre-HSCT MRD negativity (33% vs. 71%, *p* = 0.041).

Therefore, we aimed to explore a powerful strategy to eliminate pre-HSCT MRD under the guidance of MRD in the context of haplo-HSCT. Blinatumomab has been proven to be active in relapsed and refractory adult and pediatric ALL (20, 21). COG AALL1331 reported that blinatumomab has a deeper MRD clearance, improved LFS and OS, and lower toxicity compared with chemotherapy. In patients with MRD ≥0.1% after reinduction, blinatumomab was used followed by HSCT to eliminate pre-HSCT MRD in some institutes (4). However, studies on intensifying treatment with CAR-T pre-haplo-HSCT in patients with MRD ≥0.1% after reinduction are lacking. In the present study, 35.7% of patients in the chemotherapy group failed to achieve pre-HSCT MRD-negative CR, and the median level of pre-HSCT MRD was 0.3 (0%–3.8%). In the CAR-T group, 19.2% of patients failed to achieve pre-HSCT MRD-negative CR, and the median level of pre-HSCT MRD was 0.009 (0%–0.17%). Simultaneously, the CAR-T group exhibited a trend toward improved 3-year LFS and a significantly improved 3-year OS compared with the chemotherapy group (71.8% vs. 44.4%, *p* = 0.19) (84.6% vs. 40.0%, *p* = 0.008). We believe that a deeper MRD clearance effectively led to improved survival. Our data showed that MRD-based strategies, including CAR-T, improved the prognosis of relapse patients. Moreover, our study observed that patients aged <10 years had an inferior survival compared to those aged ≥10 years. Isolated bone marrow relapse was found to have inferior survival compared to bone marrow combined extramedullary relapse, which is consistent with previous studies, and may in part be due to the added TBI in patients with extramedullary relapse.

Safety was another concern regarding the application of CAR-T pre-HSCT; consistent with previous studies, no increased NRM was observed in the CAR-T group in the present study (22, 23). The median duration of engraftment after haplo-HSCT, the cumulative 100-day incidence of aGVHD grades II–IV, and the 3-year incidence of total and severe cGVHD in the CAR-T group were also similar to those of the chemotherapy group. Moreover, several studies have reported that CRS occurred in nearly 30%–94% of patients from 1 to 22 days after CAR-T cell infusion (24, 25). Maude et al. observed that 37%–78% of patients show cytopenia 1 month after CAR-T cell infusion (24). Therefore, we designed a regimen that bridged haplo-HSCT after at least 1 month of CAR-T infusion to reduce treatment-related complications. Park et al. have reported that 3 of 53 patients experienced infection-related mortality after CAR-T infusion. In the present study, no increased risk of viremia was detected in the CAR-T treatment protocol combined with haplo-HSCT.

The strengths of this study include homogeneous patient groups using consistent conditioning regimens, stem cell sources, and supportive care algorithms. However, our study has the following limitations: (1) it was not a prospective randomized study, (2) the sample size was limited, and (3) the choice of covariates for the multivariate analysis was constrained by the small number of observed events.

In conclusion, our study indicates that CAR-T therapy effectively eliminates pre-HSCT MRD, resulting in better survival in the context of haplo-HSCT. Moreover, no additional treatment-related complications were observed.

## Author Contributors

LPZ and XH designed the research and revised the paper. GH and YFC analyzed the data and wrote the paper. YZ, YJC, PS, YJ, AL, YW, SJ, LJZ, YS, CY, LX, XZ, KL, and YW collected and analyzed data. All authors contributed to the article and approved the submitted version.

## Data Availability Statement

The original contributions presented in the study are included in the article/supplementary material. Further inquiries can be directed to the corresponding authors.

## Ethics Statement

The studies involving human participants were reviewed and approved by the Peking University People’s Hospital Review Board. Written informed consent to participate in this study was provided by the participants’ legal guardian/next of kin.

## Funding

This work was supported by the Foundation of 2018 Beijing Key Clinical Specialty Construction Project-Pediatrics (2199000726) and the Foundation of CAMS Innovation Fund for Medical Sciences (CIFMS) (grant number: 2019-I2M-5-034).

## Conflict of Interest

Author YW was employed by company Beijing Yongtai Reike Biotechnology Company Ltd.

The remaining authors declare that the research was conducted in the absence of any commercial or financial relationships that could be construed as a potential conflict of interest.

## Publisher’s Note

All claims expressed in this article are solely those of the authors and do not necessarily represent those of their affiliated organizations, or those of the publisher, the editors and the reviewers. Any product that may be evaluated in this article, or claim that may be made by its manufacturer, is not guaranteed or endorsed by the publisher.
